# An insight into the estimation of drilling fluid density at HPHT condition using PSO-, ICA-, and GA-LSSVM strategies

**DOI:** 10.1038/s41598-021-86264-5

**Published:** 2021-03-29

**Authors:** S. M. Alizadeh, Issam Alruyemi, Reza Daneshfar, Mohammad Mohammadi-Khanaposhtani, Maryam Naseri

**Affiliations:** 1 Petroleum Engineering Department, Australian College of Kuwait, West Mishref, Kuwait; 2 Fouman Faculty of Engineering, College of Engineering, University of Tehran, Fouman, Iran; 3 Department of Petroleum Engineering, Ahwaz Faculty of Petroleum Engineering, Petroleum University of Technology (PUT), Ahwaz, Iran; 4 Department of Chemical Engineering, Faculty of Engineering, Golestan University, Aliabad Katoul, Iran

**Keywords:** Chemistry, Engineering, Mathematics and computing

## Abstract

The present study evaluates the drilling fluid density of oil fields at enhanced temperatures and pressures. The main objective of this work is to introduce a set of modeling and experimental techniques for forecasting the drilling fluid density via various intelligent models. Three models were assessed, including PSO-LSSVM, ICA-LSSVM, and GA-LSSVM. The PSO-LSSVM technique outperformed the other models in light of the smallest deviation factor, reflecting the responses of the largest accuracy. The experimental and modeled regression diagrams of the coefficient of determination (R^2^) were plotted. In the GA-LSSVM approach, R^2^ was calculated to be 0.998, 0.996 and 0.996 for the training, testing and validation datasets, respectively. R^2^ was obtained to be 0.999, 0.999 and 0.998 for the training, testing and validation datasets, respectively, in the ICA-LSSVM approach. Finally, it was found to be 0.999, 0.999 and 0.999 for the training, testing and validation datasets in the PSO-LSSVM method, respectively. In addition, a sensitivity analysis was performed to explore the impacts of several variables. It was observed that the initial density had the largest impact on the drilling fluid density, yielding a 0.98 relevancy factor.

## Introduction

A drilling fluid is a complicated liquid containing heterogeneous compounds of a base fluid and chemical additives. The structure of a drilling fluid should remain unchanged within the favorable temperature and pressure ranges. The drilling fluid density is an important and fundamental property in the pressure calculations of wellbores and the successful completion of drilling operations^1^. Also, downhole pressure and temperature variations significantly influence the effective density of the drilling fluid^2^. This indicates its essentiality in drilling. Decreased exploitable reserves through shallow horizons have enhanced exploration activities of larger depths^3^. In a high-pressure high-temperature (HPHT) well, the proceeding of drilling by the rise of the total vertical depth (TVD) demonstrates large alternations in the density^2,4,5^. Such alternations essentially arise from the increased bottom-hole temperature and increased mud column height in an HPHT well.

The pressure and temperature have contradictory impacts on the equivalent circulating density (ECD). In contrast, a rise in increased temperature-induced thermal expansion with the wellbore decreased ECD. These two effects are most commonly believed to balance each other^6^. However, this is not always the case, specifically concerning an HPHT well.

One can obtain the precise density alternations of drilling fluids at HPHT wells merely through real measurements^1^. To measure the density, it is required to employ precise density devices. Furthermore, the measurement procedure is difficult, costly, and time-consuming. In addition, it is not possible to derive experimental bottom-hole pressure and temperature data. Thus, it is important to develop a robust, rapid, and precise method to integrate such measurements. Intelligent approaches, e.g., the radial basis function (RBF)^7–9^, multilayer perceptron (MLP)^10–14^, LSSVM^15–19^, GA^20–22^, ICA^23–25^, and PSO^26,27^ have been of great interest to researchers to solve complicated classification and regression models in recent years. In addition, they have been employed for various petroleum and natural gas engineering purposes, e.g., the estimation of pressure–volume–temperature (PVT) characteristics, the prediction of gas characteristics, and estimation of permeability and porosity^28–33^.

The present study collected over 880 datasets, involving different mud types, temperature, pressure, and initial density (density at standard temperature and pressure), from earlier studies^34–36^. To build an efficient model, it was required to classify the data into three categories, including the training, testing and validation datasets. 75% of the real data points formed the model in the training phase, 10% of them was kept for validation phase and the remaining 15% were exploited as the testing data to perform the performance assessment of the models including PSO-LSSVM, ICA-LSSVM, and GA-LSSVM to make estimates of unobserved data. Statistic and graphical representation techniques were adopted to examine the accuracy of the model.

## Literature review

One can classify drilling fluid density prediction models into linear empirical analytical, correlation ones and intelligent approaches^37^. Many studies proposed such models to make an estimate of HPHT drilling fluid density^2,5,38^.

The impacts of the pressure and temperature in ECD estimations are of great importance^6^. Peters et al.^38^ were able to implement the compositional model of Hoberock et al.^39^ for the exploration of volumetric alternations in drilling fluids containing mineral oil/diesel as base fluids. They examined the liquid component densities of the drilling fluid at 0–15,000 psi and 78–350°F. The integration of their findings and those reported by Hoberock et al. enabled accurate predictions of drilling fluid density at HPHT condition. They derived an error of below 1% in the experimental temperature and pressure ranges. Sorelle et al.^40^ developed a less successful model on the ground of the correlations of water and hydrocarbon densities at various pressures and temperatures. Kutasov^41^ developed an analogous correlation for the prediction of density behavior of water at various temperatures and pressures, leading to accurate HPHT water densities with a significantly lower error. Isambourg et al.^5^ proposed a polynomial model of nine variables for the behavior definition of liquid drilling fluid components. The composition-grounded model, which resembled that of Hoberock et al.^39^, was found to be valid at 14.5–20,000 psi and 60–400°F. Their model assumed merely the liquid phase to be responsible for volumetric drilling fluid alternations. To employ their model, it is required to obtain the accurate reference mud density at surface conditions.

Despite their successful density modeling of drilling fluids, linear empirical correlation and analytical techniques failed to consider the impacts of the drilling fluid type on HPHT density evaluations^1^. This limits their competence in drilling fluids with particular surface densities. One can consider intelligent techniques to be a beneficial alternative to incorporate the impacts of the drilling fluid type on HPHT density evaluations. Several drilling fluid behavior models have been proposed based on artificial neural networks (ANNs) in recent years. Osman and Aggour^34^ introduced an ANN model for the prediction of the mud density based on the mud type, temperature, and pressure. The density data of drilling fluids with oil/water base fluids at 0–1400 psi and up to 400°F were exploited to train and test the ANN phases. There was a good agreement between the prediction of ANN density and experimental density measurements. Although the ANN approach was successful, such methods have a few drawbacks, including overfitting, the difficult achievement of stable solutions, a large training data requirement, and low generalizability to unobserved data^1^. Support vector machine (SVM) and LSSVM techniques may serve to solve such problems in light of their ability to solve small-sized nonlinear prediction problems and high performance for off-training set measurements^42–47^.

## Theory

### LSSVM

The LSSVM approach was introduced by Suykens and Vandewalle as a SVM variant. It is typically employed for pattern reorganization, regression, and clustering purposes^8,48,49^. The general form of LSSVM may be formulated as:

1 $$f\left( x \right) = \omega^{T} \emptyset + b$$

in which *f* relates the output (i.e., the density of mud) and input (i.e., different mud types, temperature, pressure, and initial density data). Also, $\omega$ is the weighting vector, $\emptyset$ is the mapping function, and *b* is the bias term. To estimate $\omega$ and *b*, an objective function was proposed as:

2 $$min_{\omega ,b,e} J\left( {\omega ,e} \right) = \frac{1}{2}\omega^{2} + \frac{1}{2}\gamma \mathop \sum \limits_{i = 1}^{n} e_{i}^{2}$$

3 $$y_{i} = \omega ,\phi \left( {x_{i} } \right) + b + e_{i} \quad i = 1,2, \ldots ,m$$

in which $e_{i}$ is the error of variable x_i_, and $\gamma$ is the margin parameter. One can write the regression form of LSSVM as:

4 $$f\left( k \right) = \mathop \sum \limits_{k = 1}^{N} \alpha_{k} K\left( {x,x_{K} } \right) + b$$

The present study employed the radial basis function (RBF) kernel as:

5 $$K\left( {x,x_{K} } \right) = {\text{exp}}\left( { - \frac{{x_{K} - x^{2} }}{{\sigma^{2} }}} \right)$$

in which $\sigma^{2}$ is another tuning parameter representing the squared bandwidth found by an evolutionary algorithm, e.g., genetic algorithm.

The optimization objective function is the mean square error (MSE) of LSSVM predictions^19,50^. It can be found as

6 $$MSE = \frac{{\mathop \sum \nolimits_{i = 1}^{N} \left( {MD_{pred} - MD_{exp} .} \right)}}{N}$$

in which $MD_{pred} .$ is the predicted mud density, $MD_{exp} .$ is the experimental mud density, and N is the data point count. Furthermore, one can formulate the problem as:

7 $$\min {\text{F}}({\upgamma },{\upsigma }^{2} ) = {\text{min}}\left( {{\text{MSE}}} \right)$$

### Imperialist competitive algorithms

Imperialist competitive algorithms (ICAs) are a socio-political class of strategies that have recently been adopted as an optimization approach. As with conventional optimization methods, an imperialist competitive algorithm begins with an initial population of two types of members: (1) colonies and (2) imperialists. Such members together form empires, and their competition enables optimization. A strong empire consistently attempts to control the colonies of weaker empires. Eventually, the competition results in a country with a single empire and several colonies of similar costs and positions^51^.

**Figure 1:**
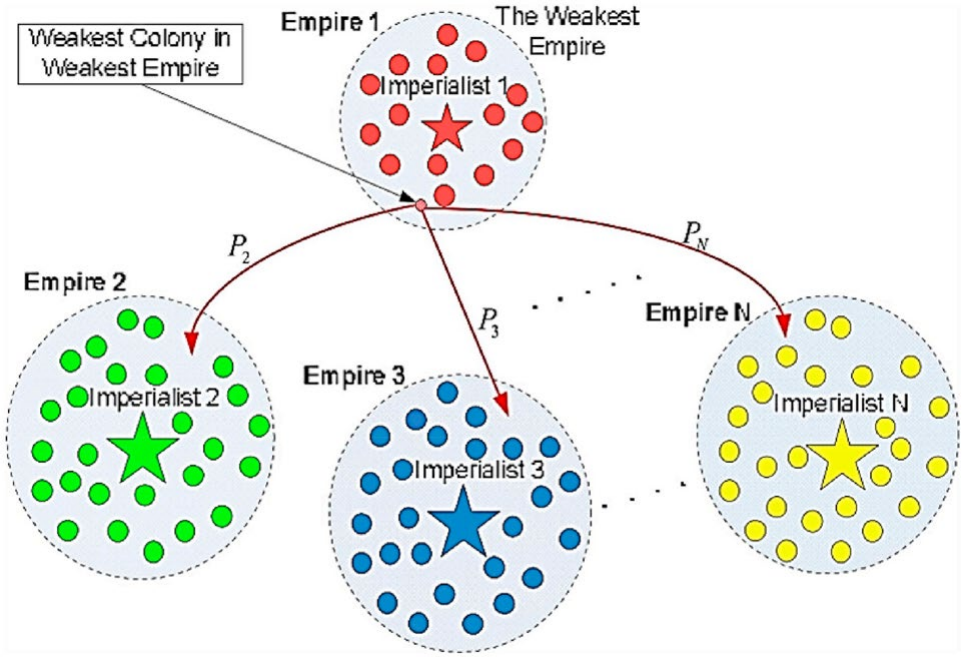
Schematic representation of the ICA optimization method^57^.

Initially, each of the imperialists owns a colony and attempts to extend their initial empire. Then, the colonies attempt to become the intended imperialistic country during evolution. Such a transformation is a policy model known as assimilation. This used to be practiced by several imperialist powers^52^. Imperialist states adopted such policies to build colonies in the favor of themselves concerning several socio-political axes, e.g., language, culture, and religion. An imperialist competitive optimization algorithm models this procedure by making the entire colonies move toward the imperialist in several optimization directions. The imperialist eventually assimilates the entire colonies. Let *d* be the imperialist-colony distance, and *x* be the travel of a colony in the direction of the imperialist^53^; Thus,

8 $$x \approx U\left( {O,\beta \times d} \right)$$

in which *x* denotes a random uniformly-distrusted number, while *β* represents a number larger than 1. In fact, *β* makes colonies approach the imperialist from the two sides. One can evaluate the overall power of the empire through the power of the imperialist state and assimilated colonies. It is obtained by summing the power of the imperialist state and a mean power portion of the corresponding colonies. The competition dissolves some empires as they are not able to win and enhance their power. As a result, greater empires continue to obtain more power every day. This leads to a state with merely one empire in the world, with the other countries being controlled by the empire state. In such a condition, the entire colonies are of the same power and position^54^. Figure 1 provides a simplified representation of an ICA. In addition to simply explaining the ICA approach, it may be proper to employ a straightforward pseudo-code to describe the ICA procedure as^55,56^:

1. Apply random points to the function to initialize the empires;
2. Move the colonies toward the corresponding imperialist (i.e., assimilation);
3. Apply random position alternations in several colonies (i.e., revolution);
4. Replace the imperialist position with a colony of a lower cost, if any;
5. Unify analogous empires;
6. Find the total cost of the empire;
7. Incorporate weaker colonies of weaker empires into a more powerful empire (i.e., imperialist competition) to exclude the empires of no power; and
8. Finish the procedure if the discontinuation criterion is met; otherwise, go to step 2.

### Genetic algorithms

Genetic algorithms (GAs) represent a popular optimization technique with a significant ability to optimize several functions. Chromosomes are the initial solution of a GA. They are randomly produced by various operators, such as mutation, crossover, and reproduction. The crossover factor (CF) and mutation factor (MF) demonstrate the alternation likelihood of the chromosomes. One can make use of CF and MF to define the offspring generation likelihood. GA steps are described as^58,59^:

1. Generating chromosomes as the initial solutions and defining CF and MF;
2. Determining the fitness of the initial solution as $F_{i}^{K} = f\left( {X_{i}^{K} } \right)$ and determining the optimal chromosome index;
3. Producing updated chromosomes by genetic operators;
4. Utilizing the fitness assessment of $F_{i}^{K + 1} = f\left( {X_{i}^{K + 1} } \right)$ to identify the best chromosome;
5. Replacing the chromosome with a new chromosome; and
6. Continue the procedure to obtain the best conditions.

**Figure 2:**
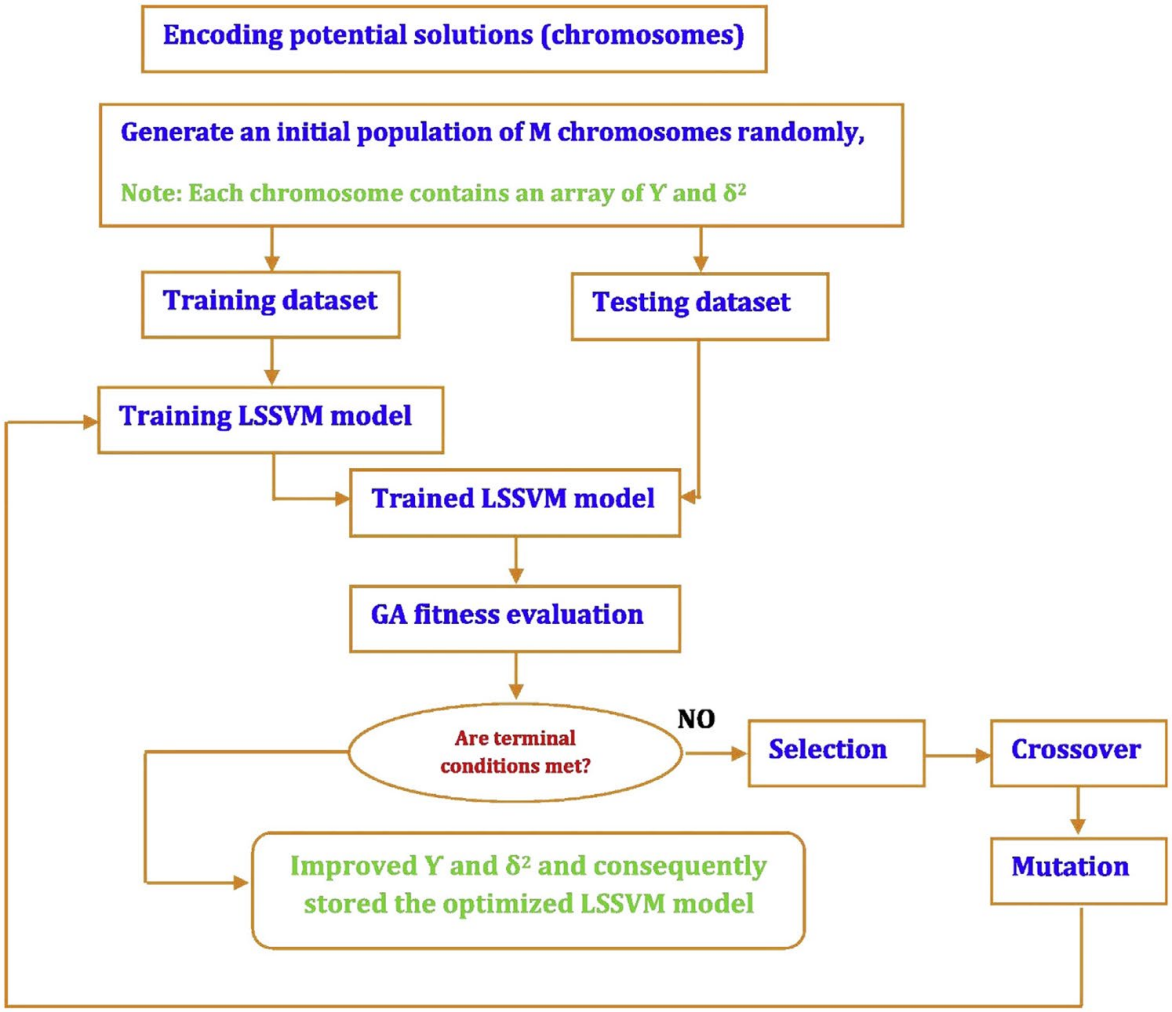
Schematic representation of the GA-LSSVM method^60^.

Figure 2 depicts the flowchart of the genetic algorithm-least square support vector machine (GA-LSSVM) approach.

### Particle swarm optimization (PSO)

Particle swarm optimization (PSO) is a stochastic optimization method grounded on various population patterns among natural species, e.g., insects, fishes, and birds^61–63^. PSO solves optimization problems by promoting initial populations^64^. Also, solutions are referred to as particles in PSO^65^. A set of particles make a swarm. The term swarm represents the population, while particles stand for individuals. Although it resembles GA in some characteristics, PSO makes use of no evolution operators such as crossover and mutation^66^. The topological particle neighborhood causes the particles to travel within the problem domain. The neighborhoods include the queen, physical neighborhood, and social neighborhood. PSO defines a velocity vector and a position vector for each of the particles^67^. The velocity of a particle is updated as:

9 $$v_{id} \left( {1 + t} \right) = wv_{id} \left( t \right) + C_{1} r_{1} \left( {P_{best,id} \left( t \right) - X_{iid} \left( t \right)} \right) + C_{2} r_{2} \left( {g_{best,id} \left( t \right) - X_{id} \left( t \right)} \right)\quad d = 1,2, \ldots D$$

where P_best,id_ is the best previous position of particle *i,* $g_{best,id}$ is the best global position of particle *i,* $w$ denotes the inertia weight, *C* is the learning rate, and *r* is a randomly-selected number. This equation involves three components, including social, cognitive, and inertia. Also, $wv_{id}$ stands for the inertia component that is the retention of the past movements and moves particle in its direction at iteration *t*. $C_{1}$ is the cognitive term and transfers the particles to their best former positions, while $C_{2}$ denotes the social component and assesses the performance of particles and the swarm trajectory within the optimization domain. The new position of a particle is indicated to be the sum of the new velocity and the previous position of the particle as

10 $$X_{id} \left( {t + 1} \right) = X_{id} \left( t \right) + v_{id} \left( {t + 1} \right) , d = 1,2, \ldots ,D$$

**Figure 3:**
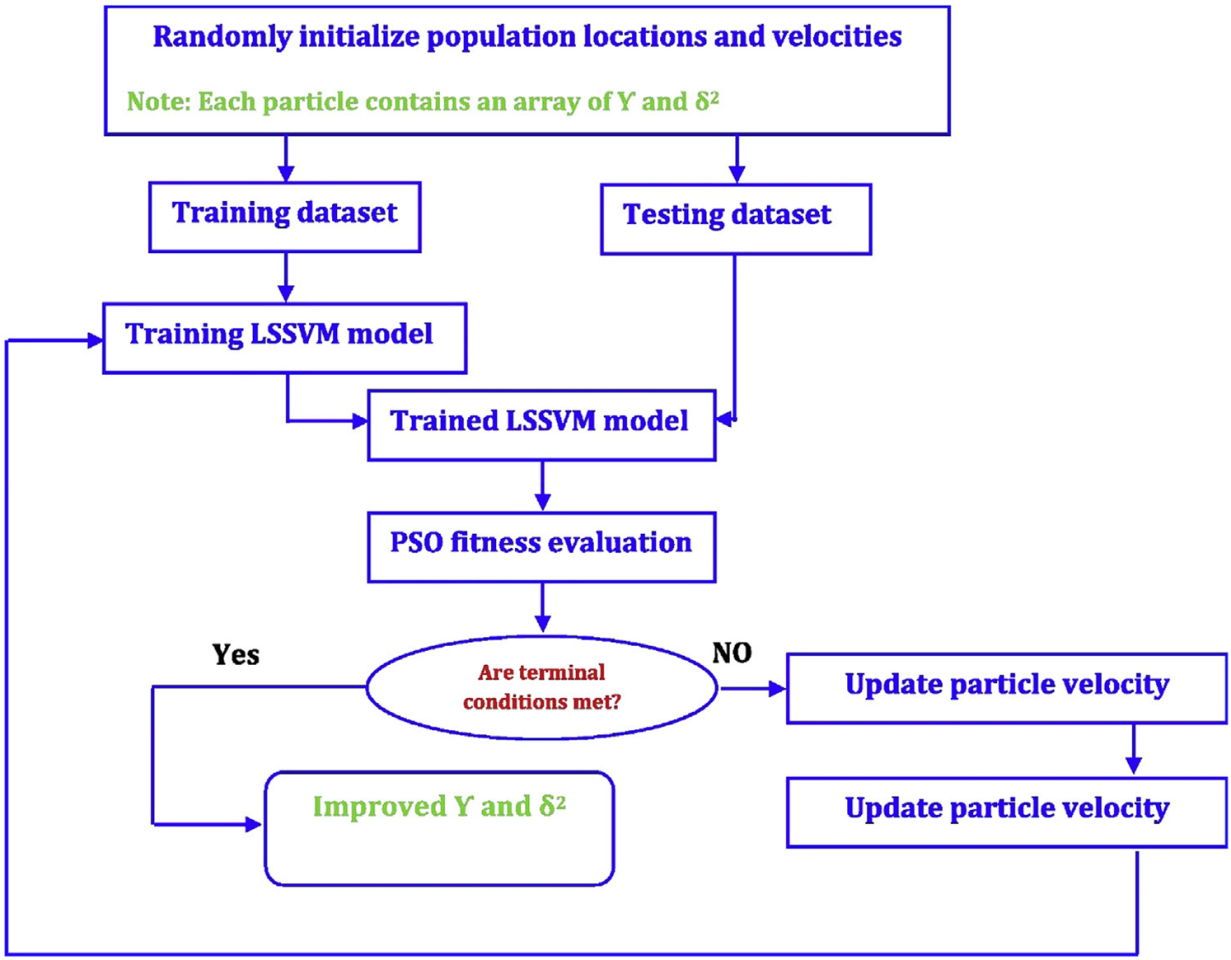
Schematic representation of the PSO-LSSVM method^68^.

Figure 3 illustrates the general form of PSO-LSSVM.

## Methodology

### Pre-analysis phase

The present work employed three analytical and modeling processes for the estimation of drilling fluid density at enhanced temperatures and pressures. The experimental findings in the first stage were utilized for model training. Four different types of liquids including water-based, oil-based, colloidal gas aphron (CGA), and synthetic fluids have been selected for the comprehensive modeling. Indices 1, 2, 3 and 4 have been used to show these drilling fluids in the model, respectively. Table 1 expresses the variation of the selected affecting parameters.

**Table 1:** Range of input affecting parameters and the output.

Parameter	Parameter range
Pressure (Mpa)	0.020252–96.98939
Type of mud	1–4
Temperature (${\text{K}}$)	294.2611–477.5944
Initial density (g/cm^3^)	0.752067–2.15698
Density (g/cm^3^)	0.629264–2.212103

Nearly 15% of the real data were exploited for model testing. Normalization was applied to the data as:

11 $$D_{k} = 2\frac{{x - x_{min} }}{{x_{max} - x_{min} }} - 1$$

in which $x$ denotes the value of parameter n. The absolute $D_{k}$ is below unity. The remaining quantities are introduced to models, building models for forecasting and validation of the density of the drilling fluid (i.e., the output). In order to model the desired process, we used MATLAB toolbox LSSVM code and coupled it with optimization codes to determine optimized weight and bias values.

### Outlier detection

The data points employed in modeling were found to be capable of posing a strong impact on the accuracy of final model estimates. Hence, the incorrect experimental data were identified by using the outlier analysis.

An outlier (or anomaly) is an essential aspect of optimization problems. In such a case, it is required to employ statistical techniques or machine learning approaches. It is suggested that outliers should be removed in a distinct stage before proceeding with the analysis . To detect outliers, the present study adopted a leverage value process. The Hat matrix was calculated as:

12 $$H = X\left( {X^{T} X} \right)^{ - 1} X^{T}$$

in which X is an N × P matrix (where N stands for the total number of data points, while P represents the number of inputs), T is the transposed operator, and -1 represents the inverse operator. A warning leverage value was defined as:

13 $$H^{*} = \frac{{3\left( {P + 1} \right)}}{N}$$

The feasible region was considered to be a rectangle restricted within 0 ≤ H ≤ H* and $- 3 \le {\text{Standard}}\,{\text{Residual}} \le + 3$. More details about this analysis are given elsewhere^69,70^.

### Model development and verification methodology

As with any model, model validation was applied in the final stage to evaluate model accuracy through a comparison of the results to the experimental data points. Validation is crucial and must undergo a revision in cases with changing variable ranges or experimental enhancements. For the development of the corresponding models, this study applied the PSO-LSSVM, ICA-LSSVM, and GA-LSSVM methods, evaluating model accuracy by statistical techniques. The accuracy quantification of the models was performed by Eqs. (14–18).

14 $$R\text{-}squared\, \left( {R^{2} } \right) = 1 - \frac{{\mathop \sum \nolimits_{i = 1}^{N} \left( {X_{i}^{actual} - X_{i}^{predicted} } \right)^{2} }}{{\mathop \sum \nolimits_{i = 1}^{N} \left( {X_{i}^{actual} - \overline{{X^{actual} }} } \right)^{2} }}$$

15 $$Mean relative \,error \,\left( {MRE} \right) = \frac{100}{N}\mathop \sum \limits_{i = 1}^{N} \left( {\frac{{X_{i}^{actual} - X_{i}^{predicted} }}{{X_{i}^{actual} }}} \right)$$

16 $$Mean\,squared\,error \left( {MSE} \right) = \frac{1}{N}\mathop \sum \limits_{i = 1}^{N} \left( {X_{i}^{actual} - X_{i}^{predicted} } \right)^{2}$$

17 $$Root\,mean\,square\,error\, \left( {RMSE} \right) = \sqrt {\frac{1}{N}\mathop \sum \limits_{i = 1}^{N} \left( {X_{i}^{actual} - X_{i}^{predicted} } \right)^{2} }$$

18 $$Standard\,deviation\, \left( {STD} \right) = \sqrt {\frac{{\mathop \sum \nolimits_{i = 1}^{N} \left( {error - \overline{error} } \right)}}{N - 1}}$$

in which X represents a property, N is the total number of data points, “actual” stands for experimental quantities, and “predicted” refers to the modeled quantities.

## Results and discussion

Table 2 reports detailed results of the models. The tuning parameters γ and σ^2^ were employed in the LSSVM method and the optimal values of them are provided.

**Table 2:** Detailed information about trained models.

Parameter	GA	PSO	ICA
σ^2^	0.951	0.648	0.781
γ	976.335	1755.632	895.644

### Model validation results

**Figure 4:**
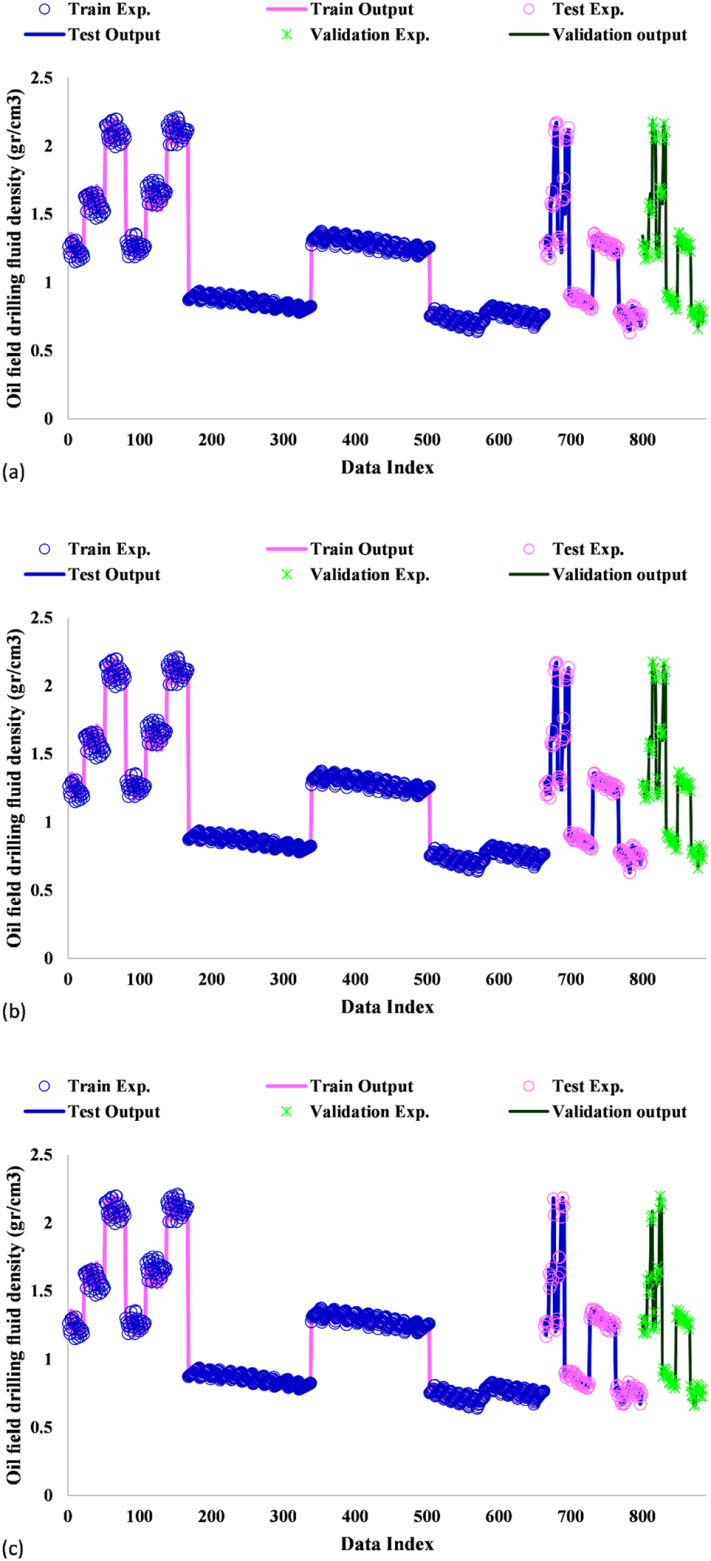
A comparison between the drilling fluid density obtained by (**a**) GA-LSSVM, (**b**) ICA-LSSVM, and (**c**) PSO-LSSVM models and the experimental values at the training, validation and testing phases.

The present work employed statistical and graphical techniques for the performance evaluation of the models concerning the prediction of drilling fluid density at HPHT condition. The results of modeled drilling fluid density are demonstrated in Fig. 4. The predictions are plotted versus the data index to represent the training, testing and validation outcomes. As can be inferred from Fig. 4, the ICA-LSSVM and PSO-LSSVM approaches yielded more satisfactory predictions as they were more accurate.

**Figure 5:**
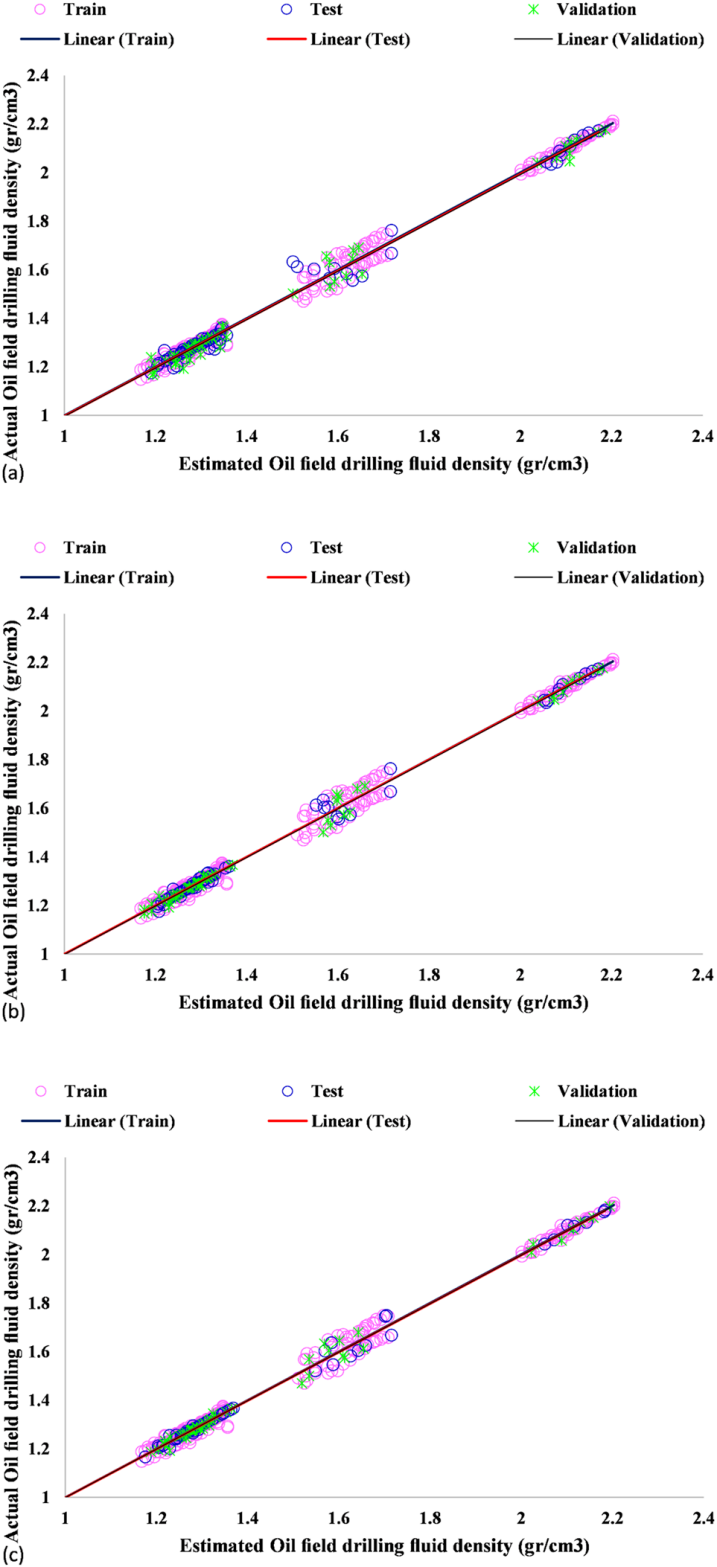
Regression plots for the prediction of drilling fluid density using (**a**) GA-LSSVM, (**b**) ICA-LSSVM, and (**c**) PSO-LSSVM models in the training, validation and testing stages.

The coefficient of determination (R^2^) demonstrates the closeness of predictions to experimental data. It ranges from 0.0 to 1.0. A coefficient of determination close to 1 stands for higher prediction accuracy. This coefficient was found to be nearly 1 for the proposed frameworks, suggesting that they had a high prediction capability for the drilling fluid density. Figure 5 illustrates the regression results of experimental and numerical coefficient of determination. As can be seen, R^2^ was found to be 0.998, 0.996 and 0.996 for the training, testing and validation datasets, respectively, in the GA-LSSVM method. R^2^ was obtained to be 0.999, 0.999 and 0.998 for the training, testing and validation datasets, respectively, in the ICA-LSSVM approach. Finally, it was found to be 0.999, 0.999 and 0.999 for the training, testing and validation datasets in the PSO-LSSVM method, respectively.

**Figure 6:**
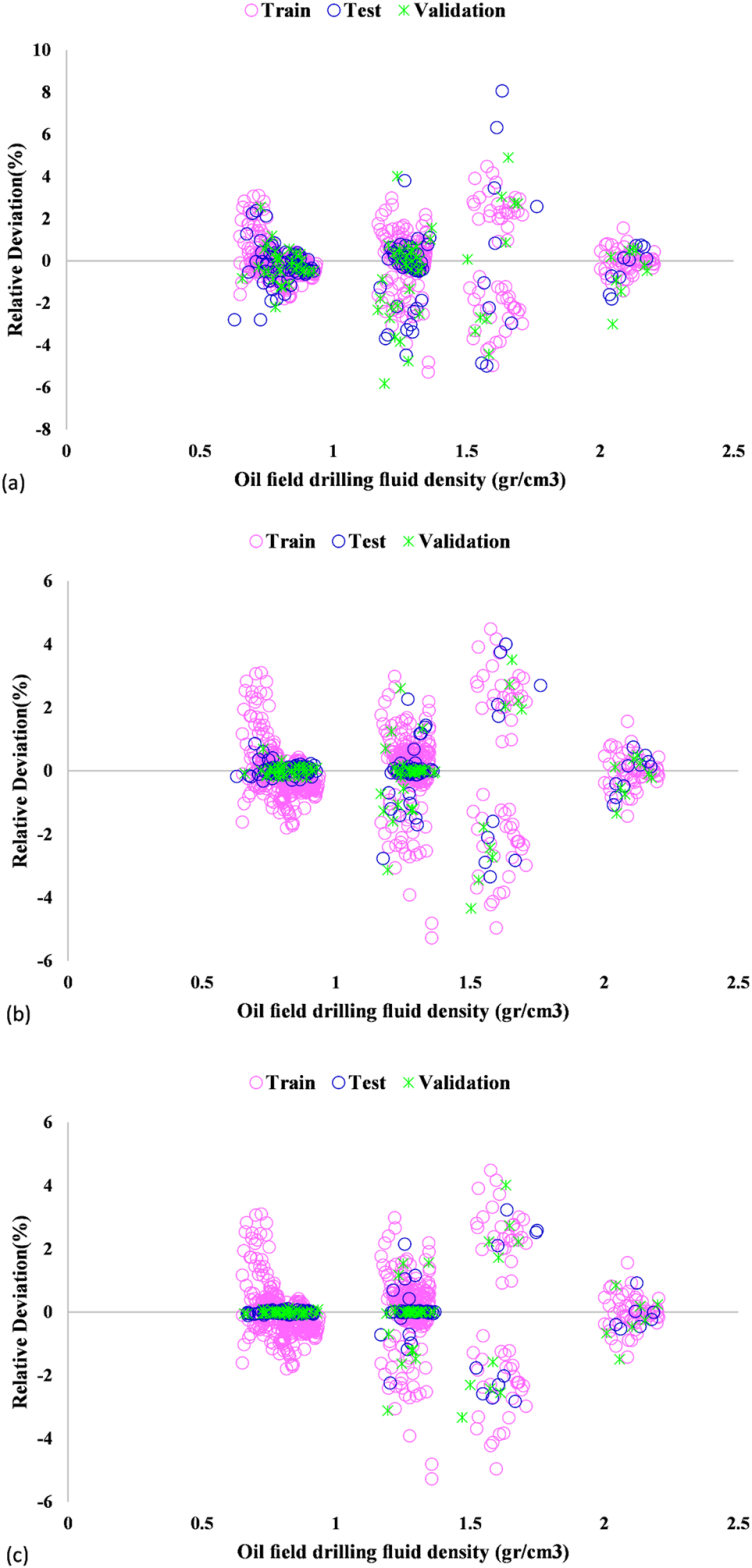
Relative deviations of the training, validation and testing datasets by (**a**) GA-LSSVM, (**b**) ICA-LSSVM, and (**c**) PSO-LSSVM.

A majority of not only the training but also testing data points were found to be distributed around the Y = X line, implying high accuracy of the model predictions. This is also true for the validation phase. Apart from Figs. 4 and 5 is supportive of the accurate predictions of the ICA-LSSVM and PSO-LSSVM techniques. Table 3 provides a detailed description of the evaluation results. According to the results, PSO-LSSVM exhibited excellent accuracy as it yielded the lowest STD, RMSE, and MRE values and the largest coefficients of determination. Figure 6 depicts the relative deviation percentages of the proposed models. The PSO-LSSVM and ICA-LSSVM models were observed to have higher accuracy as compared to the GA-LSSVM model. Also, their relative deviation did not exceed 6 percent. The relative deviation range of the GA-LSSVM model was found to be from − 8 to + 10 percent.

**Table 3:** Statistical performance evaluation of the models.

Model	Phase	R^2^	MRE (%)	MSE	RMSE	STD
GA-LSSVM	Train	0.998	0.771	0.000259006	0.0161	0.0130
	Test	0.996	1.017	0.000551279	0.023479	0.0198
	Total	0.998	0.851	0.000339246	0.0241	0.0151
	Validation	0.996	1.209	0.000625866	0.025017	0.0193
ICA-LSSVM	Train	0.999	0.464	0.000225111	0.0150	0.0134
	Test	0.999	0.455	0.000199963	0.014141	0.0126
	Total	0.999	0.478	0.000228887	0.0155	0.0135
	Validation	0.998	0.625	0.000304616	0.017453	0.0149
PSO-LSSVM	Train	0.999	0.435	0.000239871	0.0155	0.0140
	Test	0.999	0.308	0.000146462	0.012102	0.0112
	Total	0.999	0.424	0.00022778	0.0138	0.0137
	Validation	0.999	0.529	0.000263576	0.016235	0.0142

**Figure 7:**
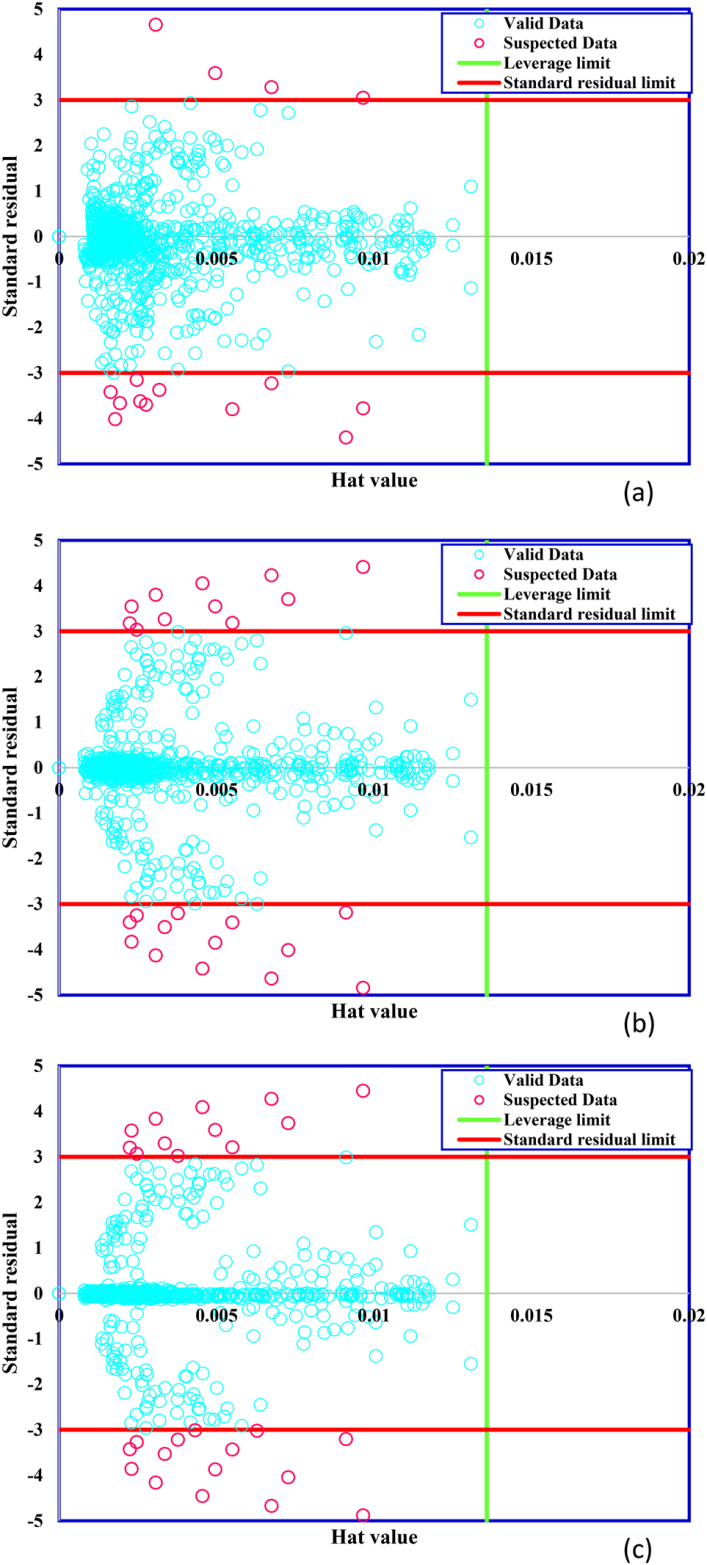
Suspicious data detection based on Hat value results applying (**a**) GA-LSSVM, (**b**) ICA-LSSVM, and (**c**) PSO-LSSVM.

The approach of adopted outlier detection was used to detect the suspicious data sets of the models, as shown in Fig. 7. According to the standard residual results versus the Hat results, this study detected 15, 27, and 27 outliers for the GA-LSSVM, ICA-LSSVM, and PSO-LSSVM models, respectively.

### Sensitivity analysis

**Figure 8:**
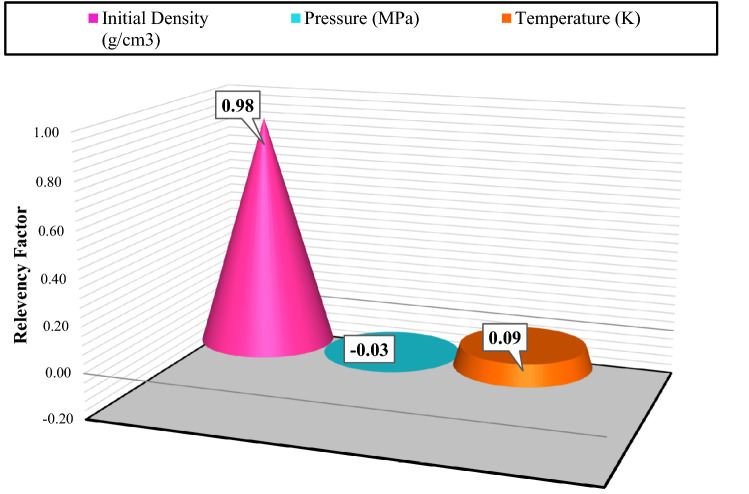
The results of sensitivity analysis for determining input impacts on the output.

A sensitivity analysis was performed to the impacts of the inputs on the output (i.e., the drilling fluid density). Then, a relevancy factor was used to calculate the quantitative impacts of the parameters as:

19 $$r = \frac{{\mathop \sum \nolimits_{i = 1}^{n} \left( {X_{k,i} - \overline{{X_{k} }} } \right)\left( {Y_{i} - \overline{Y}} \right)}}{{\sqrt {\mathop \sum \nolimits_{i = 1}^{n} \left( {X_{k,i} - \overline{{X_{k} }} } \right)^{2} \mathop \sum \nolimits_{i = 1}^{n} \left( {Y_{i} - \overline{Y}} \right)^{2} } }}$$

in which $n$ is the total number of data points, $X_{k,i}$ denotes input i of parameter k, $Y_{i}$ is output i, $\overline{{X_{k} }}$ is the average input k, and $\overline{Y}$ is the average output. The relevancy factor ranges from − 1 to + 1; a larger relevancy factor stands for a larger impact on the corresponding parameter. A positive effect indicates that arise in a particular input would raise the target parameter, while a negative impact represents a decline in the target due to an enhanced parameter. Among the input parameters, it is found that the temperature and initial density directly affected the results. At the same time, an inverse relationship was identified between the pressure and drilling fluid density; suggesting that a rise in the pressure decreases the drilling fluid density. The results of sensitivity analysis are plotted in Fig. 8. As can be seen, the pressure was found to have the strongest negative impacts, with a relevancy factor of − 0.03.

**Figure 9:**
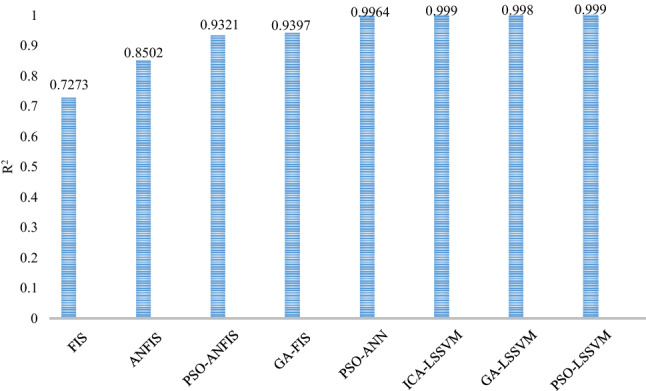
Comparison of the values of R^2^ correspond to the models proposed in this work (ICA-, GA- and PSO-LSSVM) with models available in the literature including FIS^71^, ANFIS^15^, PSO-ANFIS^15^, GA-FIS^71^, and PSO-ANN^71^.

By comparing the R^2^ values related to models developed in this study with five models found in the literature, it was concluded that the PSO-LSSVM and ICA-LSSVM models proposed in this work have the highest ability to predict the density of drilling fluid under HPHT condition. This comparison is shown in Fig. 9.

## Conclusion

The present work employed soft computing methods, including PSO-LSSVM, ICA-LSSVM, and GA-LSSVM, to model the oil field drilling fluid density at enhanced temperatures and pressures. The findings are summarized below:

- The PSO-LSSVM model yielded the most satisfactory results as it had the smallest deviation factor and highest accuracy with R^2^ and RMSE equal to 0.999 and 0.0138, respectively.
- According to our analysis, ICA-LSSVM and PSO-LSSVM exhibited higher accuracy than GA-LSSVM. Also, they did not exceed 6% in their relative deviations and GA-LSSVM was found to have a relative deviation of − 8 to 10%.
- The sensitivity analysis results demonstrated that the temperature and initial density were directly related to the drilling fluid density, while the pressure was inversely related to it.

This work could be helpful in obtaining a deeper insight into predicting the mud density and its drilling application, particularly in high performance-required applications.
